# Low-Cost In Vivo Full-Range Optical Coherence Tomography Using a Voice Coil Motor

**DOI:** 10.3390/mi13101626

**Published:** 2022-09-28

**Authors:** Xiaoqiao Liao, Liang He, Zhao Duan, Peng Tian, Yu He, Qinyuan Deng, Zeyu Ma, Ruiqi Song, Leixin Wu

**Affiliations:** 1 School of Mechanical Engineering, Sichuan University, Chengdu 610065, China; 2 Chengdu SIWI High-Tech Industrial Co., Ltd., Chengdu 610097, China; 3 State Key Laboratory of Optical Technologies for Microfabrication, Institute of Optics and Electronics, Chinese Academy of Sciences, Chengdu 610209, China; 4 School of Automation, Chongqing University of Posts and Telecommunications, Chongqing 400065, China

**Keywords:** optical coherence tomography, voice coil motor, biological microscopy, in vivo image

## Abstract

In this work, we demonstrated a novel and low-cost full-range optical coherence tomography (FROCT) method. In comparison with the off-pivot approach, which needs precise control of the deflecting distance and should be adjusted for different situations, our proposed method is more flexible without regulating the system itself. Different from the previous systems reported in the literature, which used a high-cost piezo-driven stage to introduce the phase modulation, our system utilizes a cost-effective voice coil motor for retrieving the complex-valued spectral signal. The complex-valued data, with a twofold increase in the accessible depth range, can be calculated using an algorithm based on the Hilbert transform and Dirac delta function. To confirm the effectivity of our method, both simulation and experiments were performed. In particular, for the in vivo experiment, we presented the FROCT result of a fingernail fold, demonstrating the availability of in vivo imaging. Since the key element of our system is a low-cost voice coil motor, which is flexible and more accessible for most of the clinics, we believe that it has great potential to be a clinical modality in the future.

## 1. Introduction

Optical coherence tomography (OCT), by which the two-dimensional (2D) and three-dimensional (3D) cross-section tomographic images are originated from optical low-coherence interferometry, is widely applied in ophthalmology, dermatology, gastroenterology, oncology, and interventional cardiology [1,2,3,4,5,6,7]. Due to its non-invasive characteristic, high resolution, real-time imaging and 3D tomography, OCT has rapid growth globally in both research and clinical applications. Spectral domain OCT (SDOCT), using a broadband light source and a spectrometer to measure the interference pattern as a function of wavelength, can resolve the information of tissue structure with high speed and high signal-to-noise ratio (SNR), and has become the most popular OCT modality [8,9,10]. Nonetheless, a major drawback of the SDOCT method is that the detected spectral intensity is a real function and therefore its Fourier transform is Hermitian, and the recovered image is symmetrical about the zero-path difference. Consequently, the negative and positive optical path differences with respect to the reference mirror cannot be differentiated, leading to a complex conjugate artifact that mirrors the desired, true image [11,12,13]. Generally, to avoid the mirror image caused by the mixture of negative and positive information, the sample below the zero-delay line should be purposely moved. It is well known that the system sensitivity of OCT is the highest around the zero-phase delay. Therefore, it is highly desirable to place the zero-phase delay inside the sample in order to improve the imaging contrast for highly scattering tissue [14,15,16,17,18].

In the past decade, the so-called full-range OCT (FROCT) has been proposed to eliminate the mirror terms. A number of approaches to achieve FROCT are reported, aiming to remove the ghost image and thus achieve an increased imaging depth. Initially, varieties of phase shifting methods, often applied in optical metrology, have been provided to construct complex OCT signals. Fercher A.F., et al. [12] first introduced this method into FROCT for in vitro imaging by recording five frames at the same location. Then some similar research reported the acquisition of 2–5 A-scans at a given transversal sampling location and constructing the FROCT image. These methods require high stability and are not suitable for in vivo imaging [19,20,21]. Another scheme of FROCT uses a linearly increasing phase shift generated by the uniform movement of a piezo-driven reference mirror during a B-scan, where the complex data are generated by a Hilbert transform in the transverse direction [22]. However, most of the piezo-driven translation (PZT) stages are expensive and have a limited movement range. Generally, a PZT stage with one-dimensional movement freedom has a cost higher than 1000 dollars, as the piezo-electric material used and precise control are expensive. In this case, these methods are applied with difficulty in clinical fields, even though the imaging speed is improved. Ruikang W. and Bernhard B. [23,24,25,26] demonstrated a new technology of FROCT that will not use any additional phase-shifting elements. The modulation frequency is introduced by offsetting the beam away from the pivot point when it deflects from the x-scanning mirror in the sample arm. Although these methods can achieve FROCT in a facile way, the deflecting distance from the pivot point is difficult to control and always needs to be adjusted for different situations, also causing difficulties in clinical applications. Moreover, the extinction ratio of the complex conjugate artifact heavily relies on the mirror offset and the scanning interval between two A-lines, sometimes causing the mirror artifacts to remain.

For a potential application of FROCT in clinics, herein, we proposed a new method based on a voice coil motor, which is used to introduce phase shift along each B-scan and produce the complex data. Unlike the PZT phase shifter, a voice coil motor can be more cost-effective and have a larger moving range with high stability, making it more accessible in hospitals. In this research, we developed a SDOCT system with a center wavelength of 1310 nm and added a voice coil motor in the reference arm. A modulation frequency was introduced by simultaneously moving the reference mirror while capturing the spectra data for each B-frame. Similar to the reported method [23], a Hilbert transform in the transverse direction was conducted to create the complex data, which can eliminate the mirror artifacts while applying Fourier transform. The simulation results of the multilayer structure were compiled to show the effectiveness of our proposed method. Experiments on Scotch tape and in vivo fingertips were conducted to verify that the voice coil-based FROCT can sufficiently remove the mirror artifact and achieve a deeper imaging depth.

## 2. Experimental Setup

**Figure 1:**
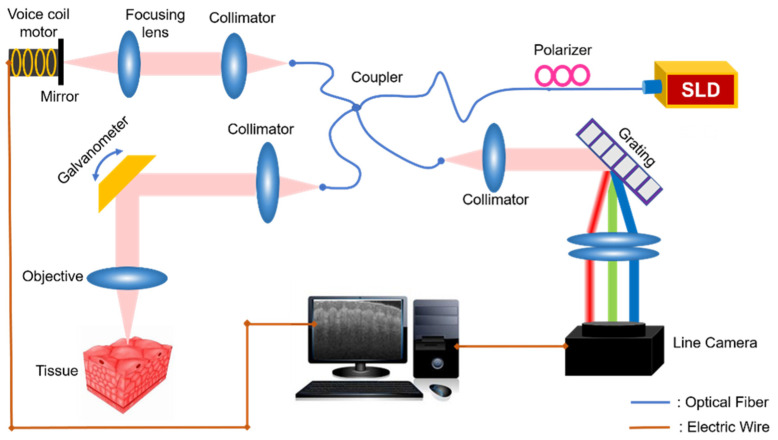
System schematic of the voice coil-based FROCT. Super luminescent diode (SLD) with a center wavelength of 1310 nm. Fiber coupler, fiber polarizer, collimator, focusing lens, galvanometer, imaging objective, reference mirror, voice coil meter and spectrometer. Spectrometer is composed of a reflection grating, a focusing lens and a line camera. The sample arm has the optical path to collect sample’s information, while the reference arm is of a moving mirror.

In our study, a voice coil-based full-range SDOCT system was developed, as shown in Figure 1. We used a near-infrared light source (Thorlabs, Newton, NJ, USA, SLD1021S) with a center wavelength of 1310 nm and a bandwidth of 70 nm. The light passes through a fiber polarizer and then comes to a fiber coupler (Thorlabs, Newton, NJ, USA, DC1300LEFA), splitting into both reference arm and sample arm. In the sample arm, the light beam is scanned by a two-axis galvanometer and finally focused on the sample by an imaging objective. In the reference arm, the reflection mirror is mounted on a voice coil motor (Moticont, Van Nuys, CA, USA, LVCM-010-013-01), whose movement is controlled synchronously with the galvanometer scanning to introduce a modulation frequency along the transverse direction. Lastly, the signals from both reference and sample arm cause interference, collected by a spectrometer (Wasatch Photonics, Logan, UT, USA, Cobra 1300) with 2048 pixels.

In FROCT of the spectral domain, by retrieving the complex-valued spectral data, the construction of OCT images will be free of conjugate artifacts caused by the unresolved signals of path length differences referring to the reference mirror. We recorded the complex-valued spectral data based on obtaining the phase of spectral interferograms as reported in reference [27]. We stored each of B-scan raw datum in a 2D matrix g(*x*,*λ*), containing 1000 A-lines, where g(*x*,*λ*) includes data points in the plane spanned by wavelength λ and transversal scanning position x. Since we simultaneously moved the voice coil motor when capturing the spectra data, a constant phase shift Δφ was introduced between each A-line. In this case, a carrier frequency, introduced along the transverse direction, was certainly helpful for removing the complex conjugate signal while applying the Hilbert transform. The complex-valued spectral data is expressed as Equation (1).

(1) $$\tilde{g}(x,\lambda )\;=\;\mathrm{HT}(g(x,\lambda ))\;\times \;\delta (x)$$

where HT is the Hilbert transform, δ(*x*) denotes the Dirac delta function. Then, the symmetrical terms of the OCT signal can be separated to construct the complex-valued spectral interferogram. It should be mentioned that the preliminary data process including background noise removal is conducted before the Hilbert transform. Next, we perform Fourier transform of $\tilde{g}$(*x*,*λ*) for each column at position *x_i_* to reconstruct the B-scan image, where the complex conjugate artifacts can be entirely removed.

## 3. Simulation and Experimental Result

**Figure 2:**
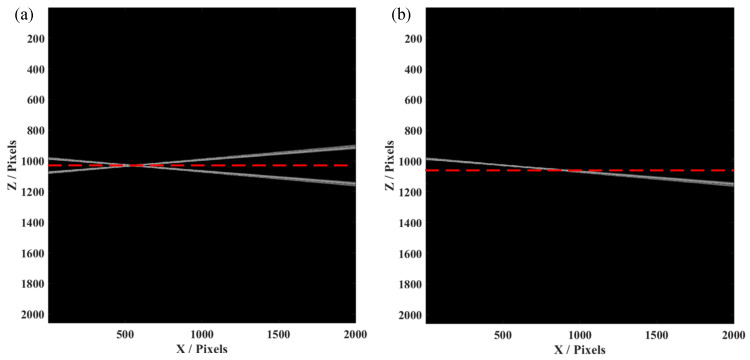
The simulation results of a single-layer structure by FROCT using a voice coil motor. (**a**) The conventional OCT image with conjugate artifacts. (**b**) The FROCT B-scan image recovered by our proposed method, is free of mirror artifacts. The red dashed line is the position of zero-phase delay. The simulated spectrometer has 2048 pixels and the value of “1024” in Y axis means the zero-phase delay position in the constructed B-scan.

Initially, we simulated an SDOCT system, whose signal to noise ratio (SNR) was 70 dB and axial resolution was 5 µm, with a center wavelength of 1300 nm. A voice coil motor, added to move the reference mirror at the sample arm, can produce a phase shift between each A-line. The spectrometer had 2048 pixels with a spectral resolution of 0.2 nm. In this case, when we constructed the conventional OCT image, only half of the pixels were effectively used, i.e., the A-line would symmetrical with the conjugate artifacts. Herein, we used a tilted mirror as the sample for measurement, which was placed to cover the two sides of the position of zero-phase delay. As shown in Figure 2a, an OCT image of the tilted mirror was constructed by the conventional data process, causing conjugate artifacts when the sample was placed over the red dashed line (zero-phase delay). This would obviously result in confusion when we observed the B-scan image, as the part of the sample seemed to be reflected. While introducing a phase shift by synchronized movement of the voice coil motor, we used the method as mentioned before to obtain the FROCT image, as shown in Figure 2b. We could clearly observe that the conjugate artifacts were efficiently removed. Thus, the total pixels could actually be used to construct the FROCT image, which can achieve a deeper imaging depth.

**Figure 3:**
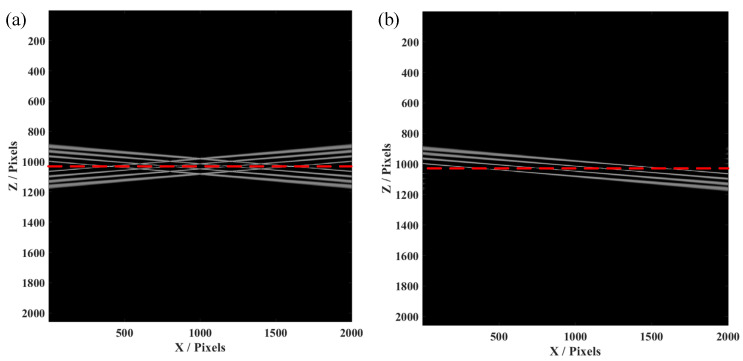
The simulation results of four-layer structure FROCT using a voice coil motor. (**a**) The conventional OCT image. (**b**) The FROCT B-scan recovered image.

Furthermore, the simulation of the four-layer structure was conducted, since the biological samples for in vivo imaging always have layered structures. This simulation on a four-layer structure is helpful for confirmation of the effectiveness and availability of our proposed FROCT method. As demonstrated in Figure 3a, conjugate artifacts are produced when the four-layer sample is placed on a red dashed line, using the same method as a single layer to obtain a FROCT image. Figure 3b indicates that the conjugate artifacts are effectively removed, achieving a deeper imaging depth. Our proposed FROCT method is validated by these simulations results.

**Figure 4:**
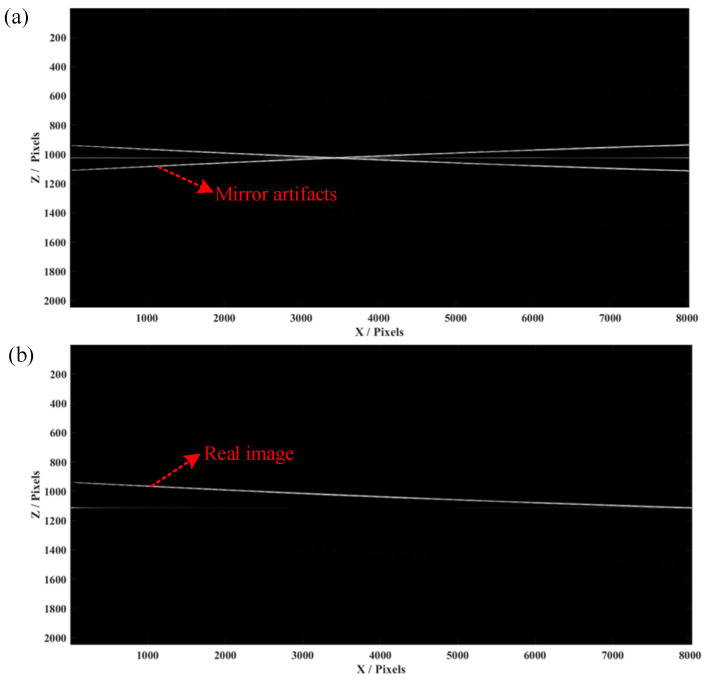
The experimental results of a mirror. (**a**) The conventional OCT image with 2048 full pixels in depth. (**b**) The FROCT image reconstructed by our proposed method using a voice coil motor.

In the experiment, we first employed a mirror and a Scotch tape as the samples with multiple layers and put the zero-phase position inside them. We set the scanning distance as 4 mm with 800 pixels and the scanning frequency as 500 Hz. The raw spectra data of all A-lines were saved in a 2D matrix, which was processed by the mentioned FROCT method. Figure 4a shows a conventional OCT algorithm to obtain a B-scan image of the mirror, indicating that conjugate artifacts are consistent with the results obtained in the single-layer simulation. A conjugate-free B-scan was obtained (Figure 4b), demonstrating a full-range image without mirroring artifacts, which matches well with the simulation results.

**Figure 5:**
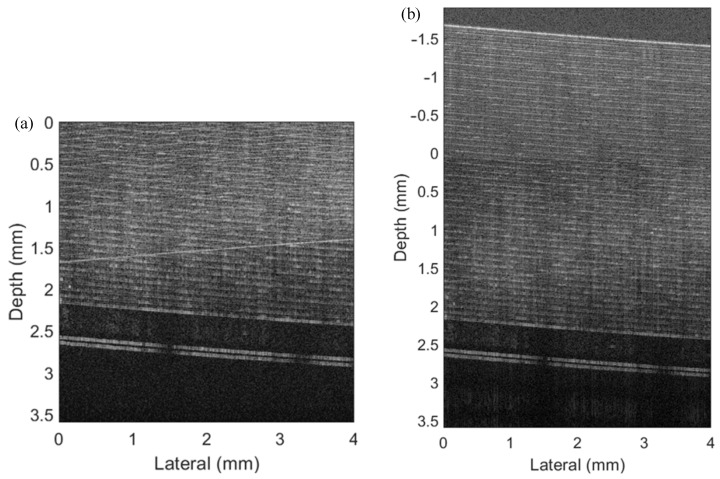
The experimental results of Scotch tape. (**a**) The conventional OCT image with conjugate artifacts, which are overlapped with the real image. (**b**) The FROCT image reconstructed by our proposed method using a voice coil motor. The FROCT image presents a larger imaging depth without mirror artifacts even though the zero-phase delay is inside the sample.

As shown in Figure 5a, we first conducted the conventional OCT algorithms to obtain the B-scan image, showing conjugate artifacts, causing overlapping information on the one side. Because the zero-phase delay was inside the sample, we could not distinguish the negative and positive parts in a normal OCT process. The conventional OCT image makes it impossible to indicate the real details of a sample once the mirror artifacts exist. Nevertheless, Figure 5b presents a conjugate-free B-scan, which shows a full-range image without mirror artifacts. The voice coil motor was moved synchronously with Galvo scanning and spectrometer capturing, to introduce a carrier frequency between A-lines. Then the numerical method was applied to reconstruct the FROCT image, from which we could observe a larger imaging range. It should be mentioned that the value “0” in the y axis illustrates the zero-phase position. The FROCT could efficiently remove the mirror artifacts and recover a clear B-scan even though the zero-phase delay was inside the sample of Scotch tape.

**Figure 6:**
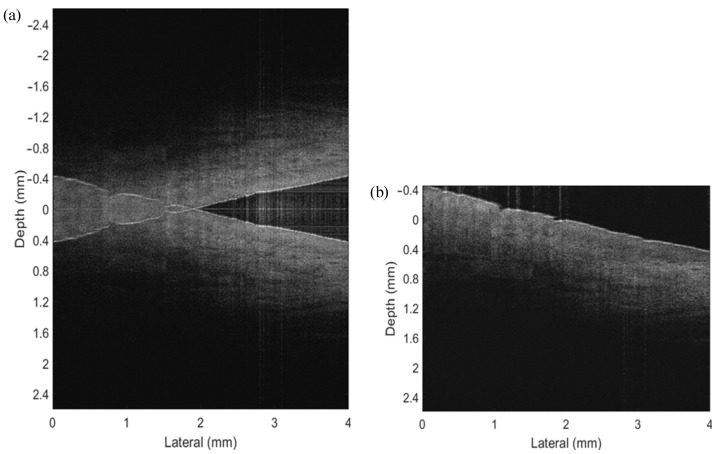
The experimental results of an in vivo nailfold on the index finger of a healthy volunteer’s right hand. (**a**) The conventional OCT image with conjugate artifacts, causing imaging confusion and overlapping of the negative and positive information. (**b**) The FROCT image reconstructed using a voice coil motor as a phase modulator. The FROCT image shows a larger imaging range with mirror artifacts removed efficiently.

To demonstrate the ability of the proposed FROCT method to image in vivo tissues, we performed an experiment on an in vivo nailfold on the index finger of a healthy volunteer’s right hand. A self-designed finger holder was put upon the sample stage to stabilize the sample while capturing the raw spectra data. Similar to the Scotch tape experiment, we set the lateral displacement as 4 mm with 500 pixels and scanning frequency as 800 Hz. The reference mirror mounted on our voice coil motor was moved synchronously with Galvo scanning and data acquisition to produce a carrier frequency along the scanning direction. With the purpose of increasing the SNR, the zero-phase delay was located inside the finger. As the standard SDOCT shown in Figure 6a, the structural details of the sample were completely deteriorated by the overlapping mirror image, making the interpretation of the OCT images almost impossible. Whereas by the application of our current approach, the structures were correctly reconstructed and the mirror terms were efficiently suppressed, as presented in Figure 6b. We concluded from the results of the in vivo nail fold that the proposed system is capable of imaging in vivo samples properly fixed on a holder.

It has been pointed out before that the sensitivity of SDOCT is highest close to the zero delay. By placing the sample across the zero-delay, it had a significant advantage in sensitivity as compared with a situation where only half of the complex space was available. In our proposed FROCT system, a cost-effective voice coil motor was used, based on a conventional SDOCT system and a carrier frequency was produced during the OCT scanning. The complex spectral data were retrieved by the Hilbert transformation and the Dirac delta function.

## 4. Discussion and Conclusions

It is well known that the system sensitivity of OCT is the highest around the zero-phase delay. Thus, it is highly desirable to place the zero-phase delay inside the sample in order to improve the imaging contrast for highly scattering tissue. Based on the principle of SDOCT, we added a cost-effective voice coil motor to produce the modulation frequency along the B-scans. The modulation frequency is used to eliminate the mirror artifacts while keeping the whole imaging depth as usual. In this way, an OCT image without overlapping areas is obtained, thus providing a clearer, large-depth image. Compared with the previous studies, our FROCT modality has lower cost and is easier to integrate with a conventional SDOCT system, as we do not use a costly PZT translation stage or offset the beam away from the pivot point when it deflects from the x-scanning mirror in the sample arm. 

In this demonstration, we first presented simulation results on the mirror sample, showing the effectiveness of our method to remove the conjugate artifacts. We purposely put the zero-delay line inside the sample, and introduced the mirror artifacts. After applying the proposed FROCT method, one could observe that the mirror errors had disappeared. Then we conducted the experiment on Scotch tape. In the conventional B-scan image, the conjugate artifacts overlapped with the real image, causing imaging errors in the construction. However, the conjugate artifacts were efficiently reduced after introducing our method. The whole imaging depth reached nearly 5 mm, which is a significant improvement in comparison with the traditional SDOCT system. Finally, we also conducted an in vivo experiment on a human finger, presenting a FROCT image with a larger imaging range. It shows that our proposed method is also applicable to in vivo tissue imaging.

By placing the sample across the zero-delay line, it has a significant advantage in sensitivity, as compared with a situation where only half of the complex space is available. The FROCT system is an efficient tool to both reduce the mirror artifacts and improve the imaging range, showing great potential for depth-resolved imaging.

In summary, we have presented a cost-effective FROCT system using a voice coil motor, aiming to introduce a phase modulation and thus produce complex spectral data. Both simulations and experiments were organized to verify our proposed method, which can efficiently suppress the complex conjugate artifacts and provide the structural details. Furthermore, in an in vivo experiment, the FROCT result of a fingernail fold was obtained, demonstrating the availability of in vivo imaging. Since the key element of our system is a low-cost voice coil motor, which is flexible for adjustment and more accessible for most of the clinics, we believe it is a highly promising way for future clinical applications in FROCT.
